# Proinflammatory and Oxidative Stress States Induced by Human Papillomavirus and *Chlamydia trachomatis* Coinfection Affect Sperm Quality in Asymptomatic Infertile Men

**DOI:** 10.3390/medicina57090862

**Published:** 2021-08-24

**Authors:** Elvia Pérez-Soto, Eduardo Fernández-Martínez, Rigoberto Oros-Pantoja, Olivia Medel-Flores, José Cruz Miranda-Covarrubias, Virginia Sánchez-Monroy

**Affiliations:** 1 Instituto Politécnico Nacional, Escuela Nacional de Medicina y Homeopatía, Gustavo A. Madero CP.07320, Mexico; elviser_1085@hotmail.com (E.P.-S.); medelflores@yahoo.com (O.M.-F.); 2 Facultad de Medicina, Universidad Autónoma del Estado de México, Toluca de Lerdo CP.50180, Mexico; rigoberto_ros@hotmail.com; 3 Centro de Investigación en Biología de la Reproducción, Área Académica de Medicina del Instituto de Ciencias de la Salud, Universidad Autónoma del Estado de Hidalgo, Pachuca CP.42090, Mexico; tomedyfm@hotmail.com; 4 Hospital Militar de Especialidades de la Mujer y Neonatología de la Secretaría de la Defensa Nacional, Miguel Hidalgo CP.11200, Mexico; hiabmiesdeaz@hotmail.com; 5 Instituto Politécnico Nacional, Escuela Superior de Medicina, Casco de Santo Tomas 11340, Mexico

**Keywords:** human papillomavirus, *Chlamydia trachomatis*, sperm quality, oxidative stress, inflammation, cytokines, male infertility

## Abstract

*Background and Objectives*: To investigate the effect of infection with human papillomavirus (HPV) or *Chlamydia trachomatis* (CT) and HPV + CT coinfection on sperm quality, inflammation, and the state of oxidative stress (OS) in asymptomatic infertile men. *Materials and Methods*: Semen samples from 84 asymptomatic military infertile men were studied. The polymerase chain reaction (PCR) was used for the molecular detection of HPV and CT. Semen parameters were analyzed according to the World Health Organization guidelines. Inflammation was evaluated by an IL-1β, IL-6, and IFN-γ enzyme-linked immunosorbent assay (ELISA) and OS by the quantification of lipid peroxidation (LPO), 8-hydroxydeoxyguanosine (8-OHdG), and total antioxidant capacity (TAC). *Results*: A total of 81 of the 84 (96.4%) samples were positives for the pathogens, with 55/81 (68%) being positive for HPV, 11/81 (13.5%) for CT, and 15/81 (18.5%) for HPV + CT coinfection. Seminal parameters were affected in the infected groups, including pH increases above the normal range in all groups. An abnormal sperm morphology was observed in the HPV and HPV + CT groups. Higher cytokine levels were detected in the HPV group and the highest IL-1β level was found in the HPV + CT group. No cytokines were detected in the CT group. High LPO and 8-OHdG levels were found in all groups with a lower TAC. Comparisons between groups showed the highest OS state was observed in the HPV group. *Conclusions*: High HPV infection or coinfection (HVP + CT) in these infertile men suggest compromising male fertility by inducing a proinflammatory state and OS. Infection with CT suggests an alteration of the state of OS by promoting an alkaline pH.

## 1. Introduction

Infections with human papillomavirus (HPV) and *Chlamydia trachomatis* (CT) are the most common sexually transmitted infections (STIs) worldwide [1,2,3], which cause considerable morbidity and socioeconomic problems [4] and may lead to death [1]. Both pathogens can present asymptomatic phases, a fact that makes the early diagnosis of male infertility challenging [1,3]. Notably, HPV infection can reduce the sperm motility [1,5] and concentration [2] and can alter normal sperm morphology [4,6]; however, the mechanisms associated with these changes have not been fully described yet. Infection with CT can cause direct damage to sperm, reducing the sperm concentration, motility [3], and vitality [1], and can increase pH [7], lipid peroxidation (LPO) [8], and even proinflammatory cytokines such as IL-1β and IL-6 in semen of infertile men [7].

Human semen contains cytokines that exert pleiotropic and redundant effects during the activation of innate, cellular, and inflammatory responses; in addition, the growth and differentiation of germ cells and functions of genital organs are regulated by proinflammatory cytokines such as IL-1β, IL-6, IL-8, TNF-α, and IFN-γ. Cytokines induced by infections, inflammation, and oxidative stress (OS) disrupt the male accessory gland function, ejaculate parameters, and sperm performance [9]. Moreover, the imbalance between reactive oxygen and nitrogen species (RONS) and antioxidants induces OS in the male reproductive tract and semen, worsening male infertility [10]. Spermatozoa are susceptible to OS-induced damage because their plasma membranes contain high concentrations of polyunsaturated fatty acids prone to LPO, generating malondialdehyde (MDA) and DNA damage and compromising fertilization [11,12]. Sperm cells are naturally protected from oxidative damage by endogenous enzymatic antioxidants in seminal plasma. Moreover, a wide variety of endogenous non-enzymatic antioxidants such as pyruvate, glutathione, carnitine, and vitamins C and E capture free radicals or RONS, which can be quantified by the total antioxidant capacity (TAC) assay [11,12]. The detection of infectious agents, inflammatory markers, and OS is necessary for the comprehensive diagnosis of male infertility [9]. The study aims to investigate the effect of HPV, CT, and HPV + CT coinfection on sperm quality, proinflammatory cytokines, and the OS state in the semen of asymptomatic military infertile men.

## 2. Materials and Methods

### 2.1. Research Design and Population

This cross-sectional study included 84 male military service members with a mean age of 31.81 ± 5.71 years; this population was selected during infertility consultation between January and November 2019 in the “Hospital Militar de Especialidades de la Mujer y Neonatología” of the National Defense Ministry, México City. Inclusion criteria were men attending the hospital for conjugal infertility investigation who failed to conceive after one year of unprotected intercourse and who agreed to be subjected to sperm parameter analysis. Men under drug therapy and those with undescended testes, varicocele, and other structural abnormalities were excluded.

### 2.2. Semen Analysis

Before semen collection and analysis, the men were asked to refrain from sexual intercourse for 3–5 days. Immediately after collection, the samples were liquefied at room temperature for up to 30 min; then, semen analysis was performed according to World Health Organization guidelines [13]. The semen samples were centrifuged at 3000× *g* for 10 min to isolate seminal plasma and cell pellets were separated and stored at −20 °C until the time of analysis.

### 2.3. Detection of HPV and Chlamydia trachomatis by PCR

Detection of HPV and *Chlamydia trachomatis*. DNA was extracted from the semen samples using the DNeasy Blood and Tissue Kit (QIAGEN, MAN, UK). The HPV genome was detected by PCR using universal primers (MY09/11, GP5+/6+ and L1C1/C2) [14]. Amplification of CT DNA was carried out using the CT-specific primers KL1 and KL2 [15]. The beta-globin gene was used as a positive control for DNA extraction and PCR methods.

### 2.4. Cytokines in the Seminal Plasma

The seminal content of proinflammatory cytokines such as IFN-γ (range: 8–3000 pg/mL), IL-1β (range: 8–1000 pg/mL), and IL-6 (range:24–1500 pg/mL) was determined using a quantitative sandwich ELISA method. Standard curves were constructed for each cytokine according to the manufacturer’s instructions (PeproTech, Rocky Hill, NJ, USA). Seminal cytokine concentrations are expressed as pg/mL of seminal plasma.

### 2.5. Assessment of Lipid Peroxidation in the Seminal Plasma

LPO was estimated in the semen samples by the measurement of malondialdehyde (MDA) using the thiobarbituric acid (TBA) method, with minor modifications [16]. Briefly, 250 μL of seminal plasma (supernatant) was added to 750 μL of 150 mM Tris-HCl, pH 7.4, in a glass tube and was incubated for 30 min at 37 °C; next, 2 mL of TBA reagent (15% trichloroacetic acid in 0.375% TBA, 1:1 *v*/*v*) was added. The final mixture was then heated for 30 min at 95 °C. After cooling to room temperature, the mixture was centrifuged at 3000× *g* for 10 min and absorbance of the supernatant was read in a spectrophotometer (Thermo Fisher Scientific, MA, USA) at 532 nm. The results were expressed as nanomoles of MDA per milligram of protein. The protein levels were quantified by the Bradford protein assay using bovine serum albumin as standard.

### 2.6. 8-Hydroxydeoxyguanosine Assay in the Seminal Plasma

DNA oxidation was evaluated by the formation of 8-hydroxydeoxyguanosine (8-OHdG), a ubiquitous marker of OS. The levels of 8-OHdG in 100 µL of seminal plasma were measured using the OxiSelect Oxidative DNA Damage ELISA Kit (Cell Biolabs Inc., CA, USA). The absorbance of each sample was determined in a microplate reader (Thermo Fisher Scientific, MA, USA) at a wavelength of 450 nm and the levels of 8-OHdG were calculated from a standard curve. The minimum detectable concentration of 8-OHdG was 20 ng/mL and the maximum detectable concentration was 100 ng/mL.

### 2.7. Quantification of Total Antioxidant Capacity

The non-enzymatic TAC of seminal plasma was measured by the ferric reducing antioxidant power (FRAP) method [16,17], with minor modifications. The FRAP working solution was prepared by mixing 10 volumes of 300 mmol/L acetate buffer, pH 3.6, with 1 volume of 10 mmol/L 2,4,6-tripyridyl-*S*-triazine (TPTZ) in 40 mmol/L HCl and with 1 volume of 20 mmol/L FeCl_3_.6H_2_O, and was warmed to 37 °C. Next, 30 µL of seminal plasma, 90 µL of distilled water, and 30 µL of each standard solution (FeSO_4_.7H_2_O; 1000, 750, 500, 250, 100 µM) were transferred to an Eppendorf tube (2 mL) and 900 µL of the FRAP working solution was added. The mixture was heated to 37 °C for 10 min. Finally, the absorbance values of blank, standard solutions, and samples were read in a spectrophotometer (Thermo Fisher Scientific, Waltham, MA, USA) at 593 nm. The results were corrected for dilution and are expressed as µmol FeSO_4_/L. All solutions were freshly prepared and immediately used. Measurements were performed in triplicate.

### 2.8. Statistical Analysis

Seminal HPV, CT, and HPV + CT coinfection are described as frequency (%). Statistical methods included mean and standard deviation (SD) for normal distribution and median (25th–75th percentile) for abnormal distribution. Specifically, the Mann–Whitney U test was used for comparison of two groups and one-factor analysis of variance (ANOVA) with post hoc Tukey or the Kruskal–Wallis one-way ANOVA was used for comparisons of more than two groups. A *p* value < 0.05 was considered statistically significant. All analyses were performed using the Statistical Package for the Social Sciences (SPSS) (version 24; SPSS Inc., Chicago, IL, USA) and Microsoft Excel (Windows 10).

### 2.9. Ethics Statement

The study protocol was approved by the institutional review board of The “Hospital Militar de Especialidades de la Mujer y Neonatología” of the National Defense Ministry, México City (CONBIOÉTICA-09-CEI-015-20180924) and informed consent was obtained from all participants. All procedures were conducted in accordance with the Declaration of Helsinki and with the Official Mexican Standard (NOM-012-SSA3-2012).

## 3. Results

**Figure 1:**
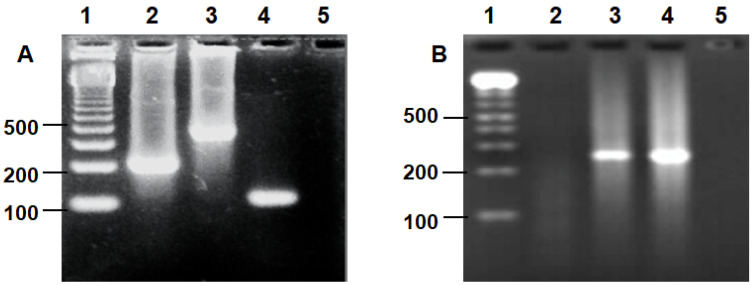
Pathogens detected in this study. Electrophoresis of PCR products from representative samples. (**A**) HPV detection. Lane 1: molecular marker 100 bp; lanes 2, 3, 4, 5: representative samples of beta-globin gene (human control), MY, GP, and L1 (L1 HPV genome), respectively (markers MY and GP PCR positive, marker L1 PCR negative). (**B**) *Chlamydia* *trachomatis* detection. Lane 1: molecular marker 100 bp; lanes 2, 3, 4, 5: representative PCR-positive (3,4) and PCR-negative (2,5) samples.

The results of the molecular assay demonstrated that 81 of the 84 samples (96.4%) were positives for the pathogens, with 55/81 (68%) being positive for HPV and 11/81 (13.5%) for CT and coinfection being detected in 15/81 (18.5%) as Figure 1.

Seminal parameters, cytokines, and OS biomarkers were analyzed and compared between the infected groups: HPV, CT, and HPV + CT (Table 1 and Table 2). An analysis of the infected groups showed that the pathogens affected seminal parameters, with the observation of pH increasing above the normal range in all groups. Abnormal sperm morphology was detected in the HPV and HVP + CT groups.

The highest cytokine levels were observed in the HPV group; however, IL-1β levels were higher in the coinfected group than in the HPV group and no cytokines were found in the CT-infected group. LPO and 8-OHdG levels were detected in all groups, in accordance with the lower TAC levels; nevertheless, the highest state of OS was found in the HPV group.

## 4. Discussion

The relevance of STIs such as those caused by HPV and CT as an etiological factor of male infertility remains controversial. Most studies report that these pathogens affect semen quality in asymptomatic infertile men [1,2,18]. Some reports suggest that sperm quality is compromised by the inflammatory processes and oxidative damage caused by CT in the male tract [1,7,8], while the role of HPV infection is still unknown. The purpose of this study was to investigate the effect of HPV, CT, and HPV + CT coinfection on sperm quality, inflammation, and the OS state in the semen of asymptomatic sexually active young men.

A high prevalence of seminal HPV infection has been demonstrated by nested PCR in oligospermic and azoospermic men (30% and 40%, respectively) [18].

This study highlighted the high prevalence of HPV infection (68%) and HPV + CT coinfection (18.5%), which is probably attributable to the specific population of military asymptomatic infertile patients analyzed who may be affected by other STIs. Other authors studying the Mexican army population found a high prevalence of seminal HPV in healthy soldiers (49%) and concluded that a risky sexual behavior, a large number of sexual partners, and anal intercourse increase the risk of acquiring and transmitting an STI [19]. Additionally, the military status contributes to the development of several psychological, endocrinological, and immunological alterations due to stress, as reported in military communities [7,20]. Such conditions can increase the susceptibility to infection. Gimenes et al. detected seven sexually transmitted agents simultaneously in men with conjugal infertility; the most prevalent STI was HPV (38.5%), followed by *Trichomonas vaginalis*, CT and HSV-1/-2 (9.6% each), highlighting that HPV + CT coinfection in the semen samples (7.1%) of asymptomatic sexually active young men (31.81 ± 5.71 years) can influence their reproductive health [21].

In this work, CT infection and CT + HPV coinfection were found in 13.5% and 18.5% of men, respectively, and the data were similar to other reports of men studied for infertility [3,21]. However, a higher prevalence (41.4%) in respect to this study was reported in young heterosexual males with chronic prostatitis-related symptoms attributable to CT + HPV coinfection [4]. Then, the early detection of pathogens such as HPV and CT may prevent the development of other diseases such as prostatitis or even prostate cancer [22].

The results suggest that semen quality was altered by the pathogens, as demonstrated by a pH increase. Recent studies suggest that infection with CT [7] and HPV [2] may cause a pH change to alkaline in the semen of infertile men, as well as shifts in the seminal microbiota, such as a decrease in *Lactobacillus* in healthy men [23]. Lactobacilli have a positive effect on sperm motility and viability [23]. The imbalance in the seminal microbiota may increase the susceptibility of patients to other infections or diseases.

According to Damke et al., HPV semen infection may modify the semen volume decrease or increase) and augment pH (≥7.8), indicating altered proportions of the fluid secreted by the major sexual accessory glands (prostate and seminal vesicles) [2]. Changes in prostate markers of glandular dysfunction may influence fertility; furthermore, HPV infection and HPV + CT coinfection altered normal sperm morphology, similar to other reports [4,6]. In addition, there is evidence that HPV infection is correlated with teratozoospermia [21].

The negative impact of HPV or CT infection on sperm morphology is not well understood. It is hypothesized that morphological disturbances may result from the ability of the virus to bind to the spermatozoa head (through interactions between HPV capsid protein L1 and syndecan-1), as reported for HPV infection [5]. Accordingly, all infected groups studied here exhibited an almost two-fold higher percentage of head defects compared to defects in the midpiece and tail; however, comparisons with an uninfected group are necessary to confirm this evidence.

Pathogens can induce the secretion of proinflammatory cytokines and OS in the semen of asymptomatic infertile men. Cytokines play a central role in physiological and pathological processes in the male reproductive tract, innate and cellular immune responses, inflammation, growth regulation, germ cell differentiation, and intracellular signal transduction [24]. Fraczek et al. hypothesized that the high concentration of proinflammatory cytokines, released during semen inflammation/infection, might modulate the activity of the pro- and antioxidative systems, provoking permanent OS in spermatozoa and reducing the fertilizing potential [24]. These bioactive substances may constitute an essential link between inflammation and male infertility [24]. The high prevalence of HPV infection or coinfection with CT in these men could have an effect of an increase in proinflammatory cytokines such as IFN-γ, IL-1β, and IL-6, with HPV mainly inducing IFN-γ and IL-6 as a proinflammatory response, probably due to the L1 protein or E6/E7 “transforming” genes found in spermatozoa [24]. HPV + CT coinfection caused a more significant proinflammatory response by the release of IL-1β; perhaps, the sum of viral proteins and CT lipopolysaccharide activate macrophages, increase RONS [24], or even cause defective spermatozoa [10].

The overproduction of RONS or reactive oxygen species (ROS) impairs sperm function [11,25]. Possibly, multiple risk factors increase the generation of ROS in sperm mitochondria [10]. Moreover, IL-1β and IL-6 might negatively influence sperm motility [25], LPO, and oxidative DNA damage by the increase in 8-OHdG [10,11,26] as the results found herein in the HPV and CT+HPV groups, reducing male fertility. Infection only with CT may generate fewer RONS, which also induces less LPO probably because antioxidant systems are still active, increasing TAC levels. However, these levels are similar to those found in other infertile populations [27] but without seminal HPV, CT, or coinfection, then other factors such as lifestyle choices (tobacco and alcohol consumption) and environmental factors trigger testicular sources of RONS [11,26], which could stimulate OS in CT infection. Moreover, seminal TAC levels were significantly lower in HPV infection or CT coinfection, resembling other infertile male populations [27], which may decrease the percentage of motile A and non-progressive motility (% motile A + B). Gholinezhad et al. found a lower percentage of motility in infertile men compared to fertile men, although with typical values of this sperm parameter according to the WHO [27].

The results suggest that infection with CT contributed to OS characterized by augmented LPO and diminished TAC levels, as reported previously [8,28]. HPV infection contributed to increased proinflammatory cytokines and the intensity of OS, which may have harmful consequences for spermatozoa, triggering alterations in sperm morphology, increasing pH, and indirectly affecting their redox profile and milieu due to the production of RONS and/or IL-1β. These molecules seem to affect the motility because fast progressive motility A was decreased, while there was a high percentage of head and tail defects in all groups; defective human sperm has been shown to spontaneously generate mitochondrial RONS to an extent that can affect sperm motility [10]. Compared to fertile men, asthenozoospermic males have reduced mitochondrial aconitase (ACO2) in spermatozoa, compromising the tricarboxylic acid cycle and, consequently, reducing the ATP synthesis and motility [29].

## 5. Conclusions

The high prevalence of HPV infection and HPV + CT coinfection detected in this study of infertile men, suggests a significant negative impact on male fertility, which could contribute to induce a proinflammatory state (increase in IFN-γ, IL-1β and IL-6), particularly an increase in IL-1β in HPV + CT coinfection. HPV or HPV + CT could induce OS by an increase in LPO and 8-OHdG and a decrease in non-enzymatic antioxidant systems, promoting an alkaline pH, alterations in sperm morphology, and decreased sperm motility A of military asymptomatic infertile men.

**Figure 2:**
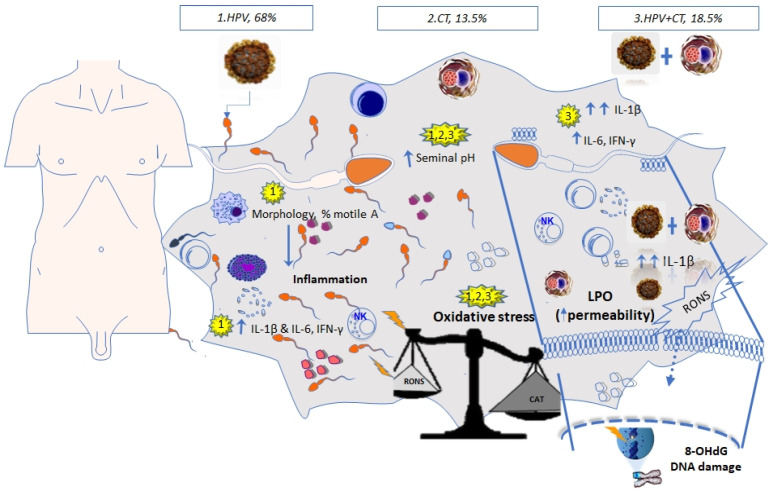
HPV infection and HPV + CT coinfection have a significant negative impact on male fertility. Pathogens induce a high proinflammatory state and oxidative stress, particularly an increase in IL-1β in HPV + CT coinfection. CT infection causes oxidative stress characterized by increased lipid peroxidation, decreased TAC levels, and increased seminal pH.

The results suggest that CT infection contributed to OS characterized by an increased LPO, decreased TAC levels, and increased seminal pH as seen in Figure 2. A comprehensive diagnosis is necessary for better treatment of male infertility and to prevent other infections or diseases in men.

The main limitation of this study included its cross-sectional design, due to it being a study of men attending the hospital for conjugal infertility investigation, and the small sample size.

Thus, a case–control study involving a more significant number of participants is required (fertile and infertile men). Studies in progress that involve healthy fertile controls of general populations and military communities could confirm the evidence obtained here. The repetition of HPV screening in semen after 12 months would be necessary to determine the clearance or persistence of seminal HPV infection. The results highlight IFN-γ, IL-1β, IL-6, LPO, 8-OHdG, and TAC evaluation in seminal plasma for a comprehensive state of male infertility to improve therapeutic intervention in male partners of infertile couples and to prevent infections or diseases.

## Figures and Tables

**Table 1:** Evaluation of seminal parameters according to infection with HPV, CT, or HPV + CT coinfection.

Parameter	HPV ^1^	CT ^2^	HPV + CT ^3^	*p*	Normal Range
N (frequency)	55/81 (68%)	11/81 (13.5%)	15/81 (18.5%)		
Age (years) †	31.40 ± 5.84	30.27 ± 4.07	33.38 ± 4.13	>0.05	
Volume (mL) †	3.29 ± 1.34 ^a^	2.19 ± 1.35	3.22 ± 0.96	<0.05	(1.5–5 mL)
pH ‡	8 (7.00–8.00)	8 (7.5–8.00)	8.00 (8.00–8.00)		(7.2–7.8)
Total sperm number (million/ejaculate) ‡	93.00 (46.00–156.00)	56.00 (32.80–148.00)	77.00 (49.00–134.00)	>0.05	(>39)
Sperm count per mL (million/mL) ‡	24 (15.00–52.00)	44.00 (15.30–97.00)	25.00 (16.00–48.00)	>0.05	(>15 million/mL)
Normal sperm morphology (%) †	2.27 ± 1.42 ^a^	5.64 ± 2.87	2.67 ± 2.58 ^c^	<0.05	(≥4%)
Abnormal sperm morphology (%) †	97.73 ± 1.42 ^a^	94.36 ± 2.87	97.33 ± 2.58 ^c^	<0.05	
Head defects (%) †	41.95 ± 8.70	43.82 ± 12.40	47.33 ± 13.71	>0.05	
Midpiece defects (%) †	25.82 ± 8.34	24.00 ± 13.19	23.27 ± 4.49	>0.05	
Tail defects (%) †	30.00 ± 12.19	26.27 ± 19.12	23.20 ± 14.30	>0.05	
Total progressive motility (% A + B) †	43.87 ± 20.00	45.00 ± 22.50	47.33 ± 18.45	>0.05	(≥32%)
Fast progressive motility (% A) †	5.96 ± 14.84	34.17 ± 28.29	2.00 ± 1.34 ^c^	<0.05	
Low progressive motility (% B) †	38 ± 18.61 ^a^	10.83 ± 23.22	45.33 ± 17.44 ^c^	<0.05	
Leukocytes (million) ‡	0.8 (0.350–1.70)	0.42 (0.110–1.48)	0.700 (0.300–1.070)	>0.05	(≤1 million)

^1^ Infertile men with human papillomavirus infection. ^2^ Infertile men with *Chlamydia trachomatis* infection. ^3^ Infertile men coinfected with human papillomavirus and *Chlamydia trachomatis*. All data are expressed as mean ± SD or median (25th–75th percentile). † ANOVA with post hoc Tukey; ‡ Kruskal–Wallis test. ^a^ HPV vs. CT group (*p* < 0.05); ^c^ CT vs. HPV + CT group (*p* < 0.05).

**Table 2:** Levels of seminal proinflammatory cytokines and oxidative stress biomarkers in the groups.

Variable	HPV ^1^	CT ^2^	HPV + CT ^3^	*p*
IFN-γ (pg/mL) ‡	524.25 (226.45–764.62) ^a^	ND	141.50 (39.00–911.90) ^c^	<0.05
IL-1β (pg/mL) ‡	141.33 (0–337.68) ^a^	0.000 (0–1.33)	328.00 (141.33–1303.00) ^b, c^	<0.05
IL-6 (pg/mL) ‡	203.30 (0.400–203.33) ^a^	ND	190.80 (36.8–271.80) ^c^	<0.05
LPO (nmol MDA/mg protein) ‡	9.00 (7.04–12.23) ^a^	4.21 (2.42–4.34)	7.45 (4.91–10.70) ^c^	<0.05
8-OHdG (ng/mL) *	8.29 (8.04–8.68) ^a^	1.9 (1.85–5.05)	8.20 (7.85–8.92)	<0.05
TAC (µmol/L) †	590.05 ± 401.11 ^a^	1086.91 ± 273.57	713.44 ± 481.11 ^c^	<0.05

^1^ Infertile men with human papillomavirus infection. ^2^ Infertile men with *Chlamydia trachomatis* infection. ^3^ Infertile men coinfected with human papillomavirus and *Chlamydia trachomatis*. Not detected, ND; interferon gamma, IFN-γ; interleukin-1 beta, IL-1β; interleukin-6, IL-6; lipid peroxidation, LPO; malondialdehyde, MDA; 8-hydroxydeoxyguanosine, 8-OHdG; total antioxidant capacity, TAC. All data are expressed as mean ± SD or median (25th–75th percentile). * Mann–Whitney U; † ANOVA with post hoc Tukey; ‡ Kruskal–Wallis test. ^a^ HPV vs. CT group (*p* < 0.05); ^b^ HPV vs. HPV + CT group (*p* < 0.05); ^c^ CT vs. HPV + CT group (*p* < 0.05).
